# Supplementary material for: Effectiveness and Safety of Ayurvedic Medicines in Type 2 Diabetes Mellitus Management: A Systematic Review and Meta-Analysis
Source: Front Pharmacol. 2022 Jun 8;13:821810. doi: 10.3389/fphar.2022.821810 (PMC9213670; doi:10.3389/fphar.2022.821810)
Supplement: Supplementary file 1 [file DataSheet1.zip › Supplementary Material/Forest and Funnel Plots/Punica granatum/Punica granatum.pdf]

Outcomes	Number of studies	Mean Diff. with 95% CI	Heterogeneity (%)
HbA1c	6	-0.06 [-0.51, 0.39]	72.93
FBG	6	-7.64 [-15.99, 0.72]	3.40
Fasting insulin*	5	-0.41 [-1.60, 0.78]	0.00
Insulin resistance	5	0.00 [-0.79, 0.80]	38.81
Body weight	4	0.78 [-2.43, 3.98]	0.00
BMI	4	0.49 [-0.42, 1.39]	1.64
Waist circumference	3	0.04 [-2.56, 2.63]	0.00
SBP	1	-11.68 [-21.32, -2.04]	N/A
DBP	1	-4.65 [-9.49, 0.19]	N/A
TC	5	-6.88 [-30.59, 16.82]	81.49
HDL-C	3	-2.09 [-8.10, 3.93]	76.94
LDL-C	3	0.21 [-18.09, 18.51]	71.10
TG	4	1.14 [-20.97, 23.25]	22.26

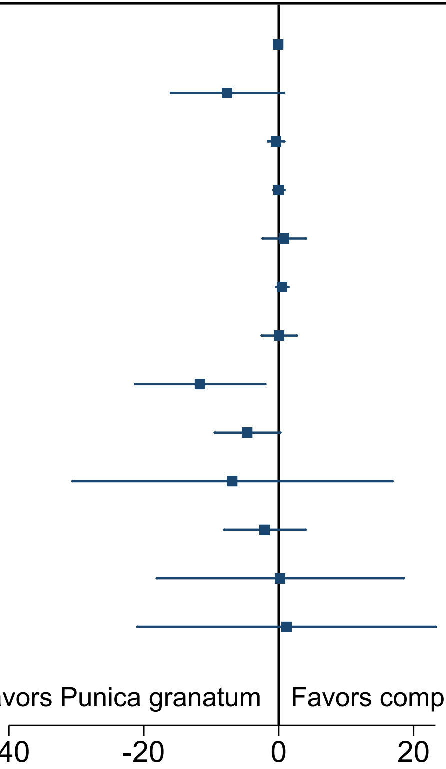

*Favors intervention/comparator as insulin sensitizer
